# Convenient Site Selection of a Floating PV Power Plant in Türkiye by using GIS-Fuzzy Analytical Hierarchy Process

**DOI:** 10.1007/s11356-024-32470-3

**Published:** 2024-02-28

**Authors:** Fatih Karipoğlu, Kemal Koca, Esra İlbahar

**Affiliations:** 1 Department of Energy Systems Engineering, Izmir Institute of Technology, Izmir, Türkiye; 2 Department of Mechanical Engineering, Abdullah Gül University, 38080 Kayseri, Türkiye; 3 KOCA Research Group, Abdullah Gül University, 38080 Kayseri, Türkiye; 4 Department of Industrial Engineering, Yıldız Technical University, Istanbul, Türkiye

**Keywords:** Floating PV systems, GIS, FAHP, Turkey

## Abstract

Floating photovoltaics (FPVs) are appearing as a promising and an alternative renewable energy opinion in which PV panels are mounted on floating platforms in order to produce electricity from renewable energy on water such as seas, dams, rivers, oceans, canals, fish farms, and reservoirs. So far, such studies related to the body knowledge on financial, technical, and environmental aspects of installation of FPV have not been performed in Turkey while expanding steadily in other countries. In this study, suitable site selection for installation of FPV power plants on three lakes in Turkey was studied by performing geographic information system (GIS) and the fuzzy analytic hierarchy process (FAHP) as multi-criteria decision-making (MCDM) method. This detailed study revealed that the criterion of global horizontal irradiance (GHI) was determined as the most crucial criterion for the installation of FPV on Beysehir Lake, Lake of Tuz, and Van Lake. Additionally, it was clearly seen that the Beysehir Lake had the highest value approximately 52% among other lakes for installation, that is why Beysehir Lake is selected as the best option for installation of an FPV system with this multi-criteria approach.

## Introduction

Recently, many countries have determined the transition from fossil fuels to renewable energy sources as a crucial step towards reducing the negative effects of climate change and addressing the energy challenges (Owusu and Asumadu 2016). Hence, new targets and strategies have been determined by governments allocating a big role to renewable energy in their gross energy consumption. In this regard, the installation of renewable energy including photovoltaic power plants and wind farms gained great reputation in the landscapes.

Particularly, energy production produced from photovoltaics and thermal systems is gradually expanding all over the world since the utilization of sunlight is free and greenhouse gasses are not released during their operation. Therefore, they are expected to play a pivotal role in terms of energy transition (Oliveira and Stokkermans 2020). On the other hand, some studies indicated a few drawbacks of solar energy technology since toxic chemicals may be released to the environment because of PV cells (Gunerhan et al. 2008; Aman et al. 2015). Furthermore, large-scale installation of a solar energy power plant requires a massive land surface. This situation may trigger loss of habitat because of deforestation and may also yield to the utilization of chemical materials to clean solar panels, bird mortality, and visual pollution (De Marco et al. 2014; Walston et al. 2016; Gasparatos et al. 2017). Therefore, novel technologies and alternative locations for installation of a solar power plant have been explored in order to overcome the constraints such as space limitation or deforestation. With this purpose, floating photovoltaic (FPV) systems have recently entered our life as a new concept in electricity production. The main objective of this recent development is to change the solar panels from ground and roof mounted types to FPV systems on inland water bodies including lakes and reservoirs. Water-based electricity production from solar power plants can lighten the pressures on land by decreasing conflicts in conjunction with other land utilization necessities. Furthermore, more energy can be revealed rather than traditional land PV panels since the evaporation process on the back of PV panels may increase lower PV cells temperature, resulting in higher efficiency (Sahu et al. 2016; Singh et al. 2019). The water loss in the reservoirs and lakes can be hindered thanks to these alternatives (Redon et al. 2014).

A considerable growth in the spread of FPV plants worldwide has been monitored over the past few years with installed capacity ranging from 10 MWp in 2014 to 2.6 GWp in 2020 (Sanchez et al. 2021). According to estimation performed by the World Bank (2019), the global potential of FPV is important (nearly 400 GWp), and numerous countries tend to invest in FPV technologies because of growing interest (Pouran 2018). Nonetheless, several aspects remain mostly understudied owing to their novelty of concept and early phase of spread and development. In particular, identification of suitable site for FPV plants deployment is an emerging requirement since FPV planners face a few challenges including the contribution to economic growth by minimizing environmental risk (e.g., visual impact) and suppressing the conflicts with other stakeholders (Kermagoret et al. 2016; Rudolph 2014).

Regarding the site selection, land-based solar PV systems were studied in the literature. One pioneered study on suitable site selection of solar PV was performed in 2011 (Charabi and Gastli 2011). They identified regions in Oman that would be suitable for the installation of solar PV plants using a GIS-based spatial fuzzy multi-criteria evaluation method. After examining nine sub-criteria and three main criteria, 0.5% of the research area was determined to be extremely appropriate for solar PV investment. A potential position for the installation of solar PV plants in Cartagena, which is located in southeast Spain, was obtained by combining the GIS and AHP methods (Sánchez-Lozano et al. 2013). Based on the analysis results, the primary consideration for selecting a solar PV site was found to be location. The two most important sub-criteria for establishing solar PV plants were determined to be the solar irradiation and the plant’s distance from the power lines. Al-Shammari et al. (2021) claimed that population density and reduction in carbon emissions had the lowest weights, while sun radiation and average temperature had the highest weights. Weights obtained by AHP analysis were used in the technique for order reported by similarity to ideal solution (TOPSIS) approach to choose the optimal site among 17 different options. Apart from studies based upon land PV power, the suitable site selection was performed for hybrid energy systems. Wu et al. (2014) created a two-stage framework for the offshore hybrid wind-photovoltaic-seawater pumped storage site selection using a hybrid MCDM technique. Four exclusion criteria were developed in order to guarantee that the natural resources of the assessed site meet the minimal requirements of the offshore hybrid wind-PV-SPS system units. A suitable site selection for hybrid renewable energy systems including wind, solar, and wave energies in the Red Sea was studied by employing the combination of GIS and AHP (Eshra and Amin 2020). The best location for installing a hybrid energy system was found to be inside Saudi Arabia’s boundaries, northwest of the Red Sea. Nyoni et al. (2021) identified hydropower reservoirs for merging hybrid-connected onshore wind and FPV systems in Zambia using a multi-criteria approach based on ranking. Based on the reservoir’s surface area, capacity factor, distance from the grid, and distance from the protected zone, 14 hydroelectric reservoirs were screened. The majority of the research for site selection in the literature was carried out by using AHP-based methodologies, and some of the research is tabulated in Table 1.

**Table 1:** Literature studies on land-/floating-based renewable energy sources

Renewable energy sources	Applied methodology	Location	References
Floating wave-wind	GIS and AHP	Greece	Vasileiou et al. (2017)
Floating wave-wind	GIS-based AHP-fuzzy OWA	China	Zhou et al. (2022)
Wind-solar PV	Ideal matter-element extension method	China	Yun-na et al. (2013)
Solar PV	GIS-based fuzzy	Turkey	Akinci and Özalp (2022)
Solar PV	GIS and AHP	Serbia	Doljak and Stanojević (2017)
Floating wind	AHP	Turkey	Caceoğlu et al. (2022)
Floating PV	GIS-based AHP and GIS-based TOPSIS	Italy	Di Grazia and Tina (2023)
Solar PV	GIS-based fuzzy	Iran	Hooshangi et al. (2023)
Floating PV	GIS	USA	Spencer et al. (2018)
Wind-solar PV	GIS and fuzzy	Mauritius	Dhunny et al. (2019)
Solar PV	GIS and AHP	Cyprus	Georgiou and Skarlatos (2016)
Solar PV	GIS	Malaysia	Sabo et al. (2016)

Apart from the studies in the literature, factors influencing location selection for conventional PV (CPV) are compared to the factors influencing location selection for FPV to assess differences between them clearly and the findings are presented in Table 2.

**Table 2:** Location influencing factors for FPV and CPV

Aspect	Impact	FPV	CPV
Stormwater infrastructure	Soil erosion	-	Necessary
Employment	Positive	Appearing	Appearing
Waste	Contamination and pollution	Required	Required
Site access	Deforestation	May exist	May exist
Site access	Traffic in the region	May increase	May increase
Bird collision with panels	Bird mortality	May exist	May exist
Sunlight blocking	Depletion of water quality	Appear on lakes	-
Deforestation	Alteration in microclimate	-	May occur
Noise	Disturb wildlife	May exist	May exist
CO_2_ saving	Positive	Existent	Existent
Energy	Positive	Existent	Existent
Visual pollution	Discomfort	May exist	May exist
Utilizing chemicals	Pollution and contamination	NA	May exist

*NA* not available

In the literature, studies on solar panel implementation were performed with various MCDM techniques including analytic network process (ANP) (Saaty 2004), AHP (Saaty 2008), TOPSIS (Hwang and Yoon 1981), fuzzy sets (Zadeh 1996), weighted aggregated sum product assessment (WASPAS) (Zavadskas et al. 2012), VIKOR (Tzeng and Huang 2011), PROMETHEE (Behzadian et al. 2010), and ELECTRE (Roy 1968). In this study, weights of the criteria, which are used as inputs in GIS, are calculated by using fuzzy AHP. AHP method has several advantages over other ranking methods. For instance, AHP method minimizes cognitive errors by making pairwise comparisons of multiple attributes. Moreover, the pairwise comparisons can be made by using not only quantitative indices but also qualitative indices (Song and Kang 2016). That is why it has been utilized in the literature for different strategic decisions such as site selection for various energy types, resource allocation, and so on. Since it is a scientific procedure with such advantages, there are several studies in the literature that utilize the outputs of AHP in GIS. For instance, both Raza et al. (2023) and Rekik and Alimi (2023) determined the suitable sites for solar and wind energy by using both AHP method and GIS, whereas Ali et al. (2023) used the combination of GIS and AHP to identify the optimal site for off-river pumped hydro energy storage. Günen (2021) and Demir et al. (2023) used both GIS and AHP methods to determine suitable sites for large-scale PV farms. In addition, Islam et al. (2023), Colak et al. (2020), and Ruiz et al. (2020) also utilized the combination of AHP method and GIS to find the optimal location for solar energy plants.

As it can be seen from the literature, AHP is a scientific procedure that is commonly utilized for different strategic decisions. If these studies are further examined, it can be seen that the size of study area is different but the combination of AHP and GIS is successfully applied. In addition, in order to obtain more precise results, fuzzy extension of AHP is utilized in this study. Fuzzy AHP provides more precise results by enabling decision makers to represent the uncertainty related to the nature of the decision-making process. For this reason, fuzzy sets are also commonly used along with GIS in the context of various energy problems on this scale in the literature (Asakereh et al. 2017; Taoufik & Fekri 2023; Sambiani et al. 2023; Gil-García et al. 2022; Dehshiri & Firoozabadi 2023; Aghaloo et al. 2023).

That is why this study utilized such a multi-criteria decision support methodology for suitable site selection of FPV power plants. Several areas for FPV installation were determined by utilizing GIS which enabled multiple layers of data by superimposing and identifying relations among them. In this respect, relative importance of evaluation criteria for FPV site selection was determined through pairwise comparisons of AHP. Because of the accurate foresight and estimation capability of installation of a power plant, GIS and AHP have been extensively employed from the past to the present especially for onshore or offshore renewables. However, there were rare studies on FPV in Turkey. Moreover, no studies have been reported about suitable site selection for FPV in Turkey. In this regard, it can be said as the novelty of this study that it will be a pioneering study in terms of installation of FPV in Turkey. Hereby, it is foreseen that this detailed study will fill a gap in the related literature by making essential contributions. Additionally, stakeholders, national authorities, and energy planners can make investment decisions and plans by using this study to identify suitable locations for FPV power plant installation.

The remainder of the paper was organized as follows: Material and methods, including the fundamental information on AHP and GIS, are presented in the “Material and methods” section. The application steps and the results of suitable site selection analysis for FPV are provided in the “Results and discussion” section. Finally, discussion and conclusions are provided in the last section of the paper.

## Material and methods

In order to determine the suitable sites for floating PV power plants by employing GIS and fuzzy AHP, initially, fundamentals on fuzzy AHP and GIS must be discussed.

### Fuzzy AHP

AHP, proposed by Saaty (1977), is one of the most commonly used MCDM methods in the literature. Different versions of this method, which can be adopted to a fuzzy environment, were introduced by Laarhoven snd Pedrycz (1983) and then by Buckley (1985). Although the calculation steps of the fuzzy AHP methods proposed in the literature might be different, the common goal of all is to perform pairwise comparisons with a scale that better represents the human judgments. In this study, the fuzzy AHP method developed by Buckley will be utilized. The difference of Buckley’s approach is that the geometric mean method is employed in the fuzzy environment while obtaining the weights of the criteria. The application steps of the fuzzy AHP method can be listed as follows (Buckley 1985; Chen and Hwang 1992):

- Step 1: A hierarchical structure is created for criteria and alternatives, and while creating this hierarchy, common criteria are grouped together as much as possible.
- Step 2: After the hierarchy is created, criteria, sub-criteria, and alternatives are evaluated by pairwise comparison using linguistic terms. In Eq. (1), the pairwise comparison matrix (*P*_*i*_) of the expert is given. The elements of this matrix, *p*_*ij*_, correspond to linguistic terms and show the importance of the *i*^th^ criterion according to the *j*^th^ criterion.

1 $${\widetilde{P}}_{i}=\left|\begin{array}{c} 1\hspace{1em}\hspace{0.33em}{\widetilde{p}}_{12} \dots \hspace{1em}{\widetilde{p}}_{1n} \\ { \widetilde{p}}_{21}\hspace{1em}1 \dots \hspace{1em}{\widetilde{p}}_{2n} \hspace{0.33em}\hspace{0.33em}\\ \vdots \hspace{0.33em}\hspace{0.33em}\hspace{0.33em} \hspace{0.33em} \vdots \vdots \vdots \hspace{1em}\hspace{0.33em}\hspace{0.33em}\vdots \\ \vdots \hspace{0.33em}\hspace{0.33em}\hspace{0.33em}\hspace{0.33em} \vdots \vdots \vdots \hspace{1em}\hspace{0.33em}\hspace{0.33em}\vdots \\ {\widetilde{p}}_{n1} \hspace{0.33em}{\widetilde{p}}_{n2}\hspace{0.33em}\dots 1\end{array}\right|$$

The pairwise comparison matrix given in Eq. (1) and consisting of linguistic data is converted into triangular fuzzy numbers (TFNs) using the scale given in Table 3. The representation of the pairwise comparison matrix with TFNs is given in Eq. (2) (Hsieh et al. 2004; Cebi and Ilbahar 2021).

**Table 3:** Linguistic scale and corresponding TFNs

Linguistic term	Abbreviation	Fuzzy number
Absolutely unimportant	AU	(1/9, 1/9, 1/7)
Very unimportant	VU	(1/9, 1/7, 1/5)
Unimportant	U	(1/7, 1/5, 1/3)
Slightly unimportant	SU	(1/5, 1/3, 1)
Equally important	EI	(1, 1, 3)
Slightly important	SI	(1, 3, 5)
Important	I	(3, 5, 7)
Very important	VI	(5, 7, 9)
Absolutely important	AI	(7, 9, 9)

2 $${\widetilde{P}}_{i}=\left|\begin{array}{c} 1\hspace{1em}\hspace{0.33em} \left({p}_{12}^{l}, {p}_{12}^{m},{p}_{12}^{u}\right)\hspace{1em}\dots \hspace{1em}\left({p}_{1n}^{l}, {p}_{1n}^{m},{p}_{1n}^{u}\right)\\ \left({p}_{21}^{l}, {p}_{21}^{m},{p}_{21}^{u}\right)\hspace{1em} 1\hspace{1em}\hspace{0.33em} \dots \hspace{1em}\left({p}_{2n}^{l}, {p}_{2n}^{m},{p}_{2n}^{u}\right)\\ \vdots \hspace{1em}\hspace{0.33em} \vdots \hspace{0.33em} \vdots \hspace{0.33em}\hspace{0.33em} \vdots \\ \left({p}_{n1}^{l}, {p}_{n1}^{m},{p}_{n1}^{u}\right)\left({p}_{n2}^{l}, {p}_{n2}^{m},{p}_{n2}^{u}\right) \dots 1\end{array}\right|$$

Step 3: After the consistency analysis of these pairwise comparison matrices are made, a co-decision matrix is obtained by using aggregation operations. After calculating the fuzzy geometric mean value for each row of the co-decision matrix as in Eq. (3), the fuzzy weights are calculated with the help of Eq. (4).

3 $${\widetilde{r}}_{i}=({\widetilde{p}}_{i1}\otimes {\widetilde{p}}_{i2}\otimes ...\otimes {\widetilde{p}}_{in}{)}^{1/n}$$

4 $${\widetilde{w}}_{i}={\widetilde{r}}_{i}\otimes ({\widetilde{r}}_{1}+{\widetilde{r}}_{2}+...+{\widetilde{r}}_{n}{)}^{-1}$$

By using the fuzzy weights obtained in this step, the calculation procedure can be continued, as well as the crisp weights can be obtained by using the defuzzification formula given in Eq. (6).

Step 4: In this step, the fuzzy weights obtained in step 3 and the fuzzy performance evaluations of the alternatives are combined using Eq. (5), and the total scores of the alternatives are obtained.

5 $${\widetilde{S}}_{i}={\sum }_{j=1}^{n}{\widetilde{w}}_{j}{\widetilde{r}}_{ij}, \forall i$$

Step 5: The total scores obtained as fuzzy numbers of the alternatives are defuzzified using Eq. (6).

6 $${NS}_{i}=\frac{\left({S}_{i}^{u}-{S}_{i}^{l}\right)+\left({S}_{i}^{m}-{S}_{i}^{l}\right)}{3}+{S}_{i}^{l}, \forall i$$

Step 6: By normalizing the total scores obtained using Eq. (7), the best alternative can be decided (Cebi and Ilbahar 2021).

7 $${NS}_{i}^{n}=\frac{{NS}_{i}}{{{\sum }_{i=1}^{N}NS}_{{\text{i}}}}, \forall i$$

### Geographical information systems

GIS is a tool to visualize the data, and it has gained more reputation in the last decade in energy projects. For solving complex problems of suitable site selection, GIS could be a suitable way to proceed (Öztürk and Karipoğlu 2022a, 2022b). As it can be used to examine the effect of several geographic criteria, GIS is intensively used for the determination of suitable regions. This study used the GIS software as a main analysis tool, and the ArcGIS version was utilized to investigate the suitable locations for FPV.

In order to obtain the suitability map layer, the data processed in the GIS software should be categorized by assigning the same points. Thus, the detection of best and worst suitable regions can be done easily. The rationale of scoring stated by the reviewer is carried out by following the four steps: (i) the determination of the range of data obtained; (ii) the detection of the maximum and minimum points; (iii) if available, the determination the buffer zone of each criterion from literature; and (iv) the completion of the scoring distribution. Even if the numbers of classifications seem different in the literature, the general preference is to use five classifications (Rahmat et al. 2017; Al Garni and Awasthi 2017; Noorollahi et al. 2016; Alkaradaghi et al. 2019; Genç et al. 2021; Elboshy et al. 2022; Yılmaz et al. 2023). The five classifications (from 0 to 10) are suitable to make the suitability index very low, low, meditate, high, and very high for different locations.

### Possible study areas

**Fig. 1:**
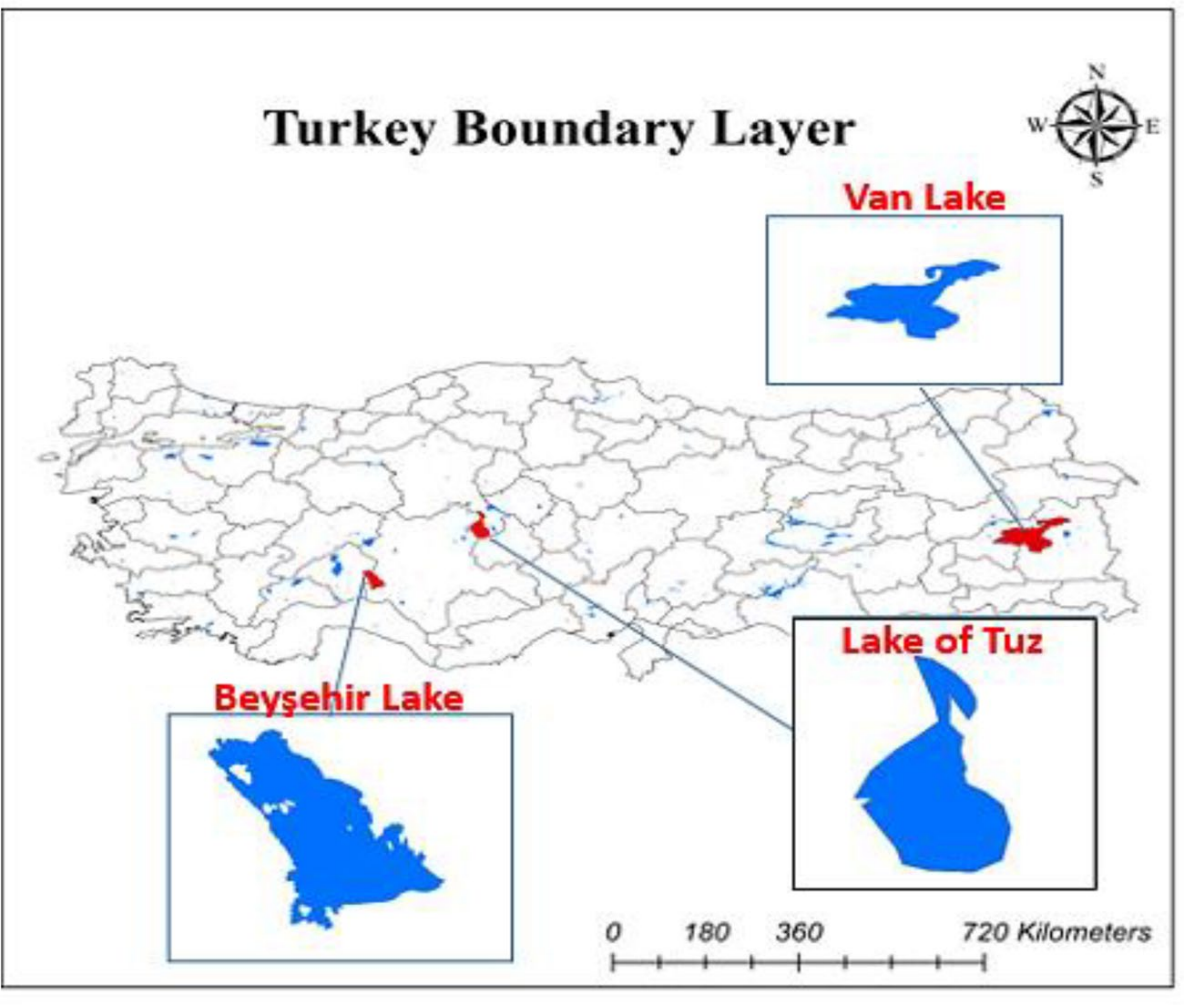
Geographical locations of study areas

Turkey has improved as a developing country in terms of renewable energy systems. Onshore wind and solar power plants have shown great improvement in the last decade. But there is not offshore energy application in Turkey since it has huge seas and water bodies (Genç et al. 2021). This study investigated the suitability of three lakes which are selected from water bodies for FPV power systems. Figure 1 shows the water bodies in Turkey and selected study areas named as Beysehir Lake, Tuz Lake, and Lake of Van. The reason for selecting these areas is to investigate the environmental impacts of different criteria while considering the same daylight zone.

## Results and discussion

This section presents the determined suitable or non-suitable region in the study areas based on the combination of GIS and fuzzy analytic hierarchy process (FAHP) results. Under the three main criteria, ten critical sub-criteria of the suitable site selection for FPV systems were considered. According to FAHP results, global horizontal irradiance (GHI) criteria was determined as the most important restriction for the potential FPV applications. Therefore, the technical impact has more critical importance than environmental and accessibility criteria. These weights obtained by FAHP analysis showed that the investigation of technical criteria for FPV systems is a vital step to be done by energy planners or engineers.

According to weights of each sub-criterion (except for human activities and marine effect), all map layers were overlaid in GIS. The resulting final suitability map is presented in Fig. 9 with suitability scores for each lake. The results show that Beysehir Lake has approximately 273 km^2^ region suitable for FPV, whereas the most suitable region covers an area of 1159 km^2^ in Van Lake. Lake of Tuz has about 144 km^2^ suitable regions which consist of 26% of the surface area of Lake of Tuz. Of the most suitable regions with the total area of lakes, the Beysehir Lake had the highest value with nearly 52%. Therefore, Beysehir Lake could be a good area for the first FPV application in Turkey.

### Application of FAHP

In this study, the criteria identified for the installment of FPV systems were evaluated by eight experts with at least 2 years of experience in the field of solar energy. Six of the experts majored in energy systems engineering, while the other two majored in environmental engineering. After these experts individually filled the pairwise comparison matrices using linguistic terms, these terms were converted into corresponding triangular fuzzy numbers and aggregated while analyzing their consistency. Tables 4, 5, 6, and 7 show the aggregated version of pairwise comparison matrices for main criteria, technical criteria, environmental-social criteria, and accessibility criteria, respectively. After Table 4, 5, 6, and 7 were constructed, the steps of FAHP were implemented and the fuzzy weights of these criteria and sub-criteria were calculated as given in Table 8. Then, these fuzzy weights, represented with TFNs, were defuzzied and final weights of these sub-criteria were obtained. The final weights are presented in Table 8, and according to this table, the GHI was found as the most important sub-criteria with nearly 0.47, while the restriction of human activities had the lowest importance weight. These final weights were used as input in GIS to evaluate different lakes in Turkey with respect to their suitability for FPV systems. Even though the map layers could not be obtained for human activities and possible negative effects to marine due to the lack of proper data for these restrictions, based on the FAHP results, they do not affect the results dramatically since the total importance weight of both criteria is about 0.12.

**Table 4:** Aggregated pairwise comparison of main criteria for solar FPV systems

Main criteria	Technical criteria	Environmental and social criteria	Accessibility criteria
Technical criteria	(1.00, 1.00, 1.00)	(2.37, 3.00, 5.52)	(2.14, 3.50, 5.81)
Environmental and social criteria	(0.18, 0.33, 0.42)	(1.00, 1.00, 1.00)	(0.53, 0.80, 2.14)
Accessibility criteria	(0.17, 0.29, 0.47)	(0.47, 1.25, 1.90)	(1.00, 1.00, 1.00)

**Table 5:** Aggregated pairwise comparison of technical criteria for solar FPV systems

Technical criteria	Global horizontal irradiance	Elevation	Surface area
Global horizontal irradiance	(1.00, 1.00, 1.00)	(4.36, 6.43, 8.14)	(4.43, 6.87, 8.00)
Elevation	(0.12, 0.16, 0.23)	(1.00, 1.00, 1.00)	(1.00, 1.25, 3.32)
Surface area	(0.12, 0.15, 0.23)	(0.30, 0.80, 1.00)	(1.00, 1.00, 1.00)

**Table 6:** Aggregated pairwise comparison of environmental and social criteria for solar FPV systems

Environmental and social criteria	Human activities	Distance to protected areas	Distance to forest area	Water pollution/marine effect
Human activities	(1.00, 1.00, 1.00)	(0.58, 0.73, 1.74)	(0.76, 1.18, 2.36)	(0.24, 0.35, 0.68)
Distance to protected areas	(0.57, 1.37, 1.72)	(1.00, 1.00, 1.00)	(1.47, 2.27, 4.58)	(0.36, 0.46, 1.17)
Distance to forest area	(0.42, 0.84, 1.32)	(0.22, 0.44, 0.68)	(1.00, 1.00, 1.00)	(0.16, 0.23, 0.47)
Water pollution/marine effect	(1.47, 2.89, 4.21)	(0.85, 2.17, 2.76)	(2.11, 4.37, 6.28)	(1.00, 1.00, 1.00)

**Table 7:** Aggregated pairwise comparison of accessibility criteria for solar FPV systems

Accessibility criteria	Distance to electrical grid	Distance to main roads	Distance from land
Distance to electrical grid	(1.00, 1.00, 1.00)	(3.41, 5.04, 7.06)	(2.24, 3.04, 5.54)
Distance to main roads	(0.14, 0.20, 0.29)	(1.00, 1.00, 1.00)	(0.51, 0.68, 1.73)
Distance from land	(0.18, 0.33, 0.45)	(0.58, 1.46, 1.97)	(1.00, 1.00, 1.00)

**Table 8:** Fuzzy weights and final weights of assessment criteria

Criteria	Fuzzy weights	Final weights
**Technical criteria**	(0.34, 0.62, 1.22)	
Global horizontal irradiance	(0.48, 0.77, 1.14)	0.468
Elevation	(0.09, 0.13, 0.26)	0.093
Surface area	(0.06, 0.11, 0.17)	0.066
**Environmental and social criteria**	(0.09, 0.18, 0.37)	
Human activities	(0.09, 0.16, 0.45)	0.033
Distance to protected areas	(0.11, 0.24, 0.56)	0.043
Distance to forest area	(0.05, 0.12, 0.27)	0.021
Water pollution/marine effect	(0.16, 0.49, 1.18)	0.087
**Accessibility criteria**	(0.08, 0.20, 0.37)	
Distance to electrical grid	(0.38, 0.66, 1.19)	0.125
Distance to main roads	(0.08, 0.14, 0.28)	0.028
Distance from land	(0.09, 0.21, 0.34)	0.036

### Criteria assessment and data sources

This study considers the technical, environmental, and social as well as accessibility criteria as main criteria. Also, ten sub-criteria under main criteria were examined and discussed for their possible effects on FPV systems. Table 9 demonstrates the used main/sub-criteria, reclassified/score, and importance values that are used in GIS. To examine the effect of these sub-criteria, proper and updated data sources such as Global Solar Atlas (2020), Copernicus Land Monitoring (2018), OpenStreet (2022), Turkey Electric Transmission Company (TEİAS 2019), and Natura (2000) were utilized.

**Table 9:** The list of sub-criteria and the corresponding units and suitability scores

Main criteria	Sub-criteria	Unit	Reclassified/score	Importance value
***C1. Technical***	Global horizontal irradiance	*kWh/m*^*2*^	< 1300 / **0** 1300–1400 / **2** 1400–1450 / **4** 1450–1500 / **6** > 1550 / 1**0**	0.468
	Elevation	*m*	> 2500 / **0** 1500–2000 / **4** 1000–1500 / **6** 1000–500 / **8** > 500 / **10**	0.093
	Surface area	*m*^*2*^	< 500 / **0** 500–1000 / **2** 1000– 1500 / **4** 1500–3000 / **6** > 4000 / **10**	0.066
***C2. Environmental***	Dist. from forest areas	*m*	< 200 / **0** 200–800 / **2** 800–1200 /** 4** 2000–3000 / **8** > 3000 / **10**	0.021
	Dist. from protected areas	*km*	< 5 / **0** 5–10 / **2** 10–15 / **4** 15–20 / **6** > 25 / **10**	0.043
	Human activities	*-*		0.033
	Water pollution/marine effect	*-*		0.087
***C3. Accessibility***	Dist. from electrical grid	*km*	> 50 / **0** 40–50 / **2** 30–40 / **4** 10–20 / **6** < 10 / **8**	0.125
	Dist. from main roads	*km*	> 50 / **0** 40–50 / **2** 30–40 / **4** 10–20 / **8** < 10 / **10**	0.028
	Dist. from land	*m*	< 100 / **0** 100–200 / **2** 200–300 / **4** 300–400 /** 6** > 500 / **10**	0.036

The significance of the bold emphasis increasing suitability scores ranging from 0 to 10

#### GHI

**Fig. 2:**
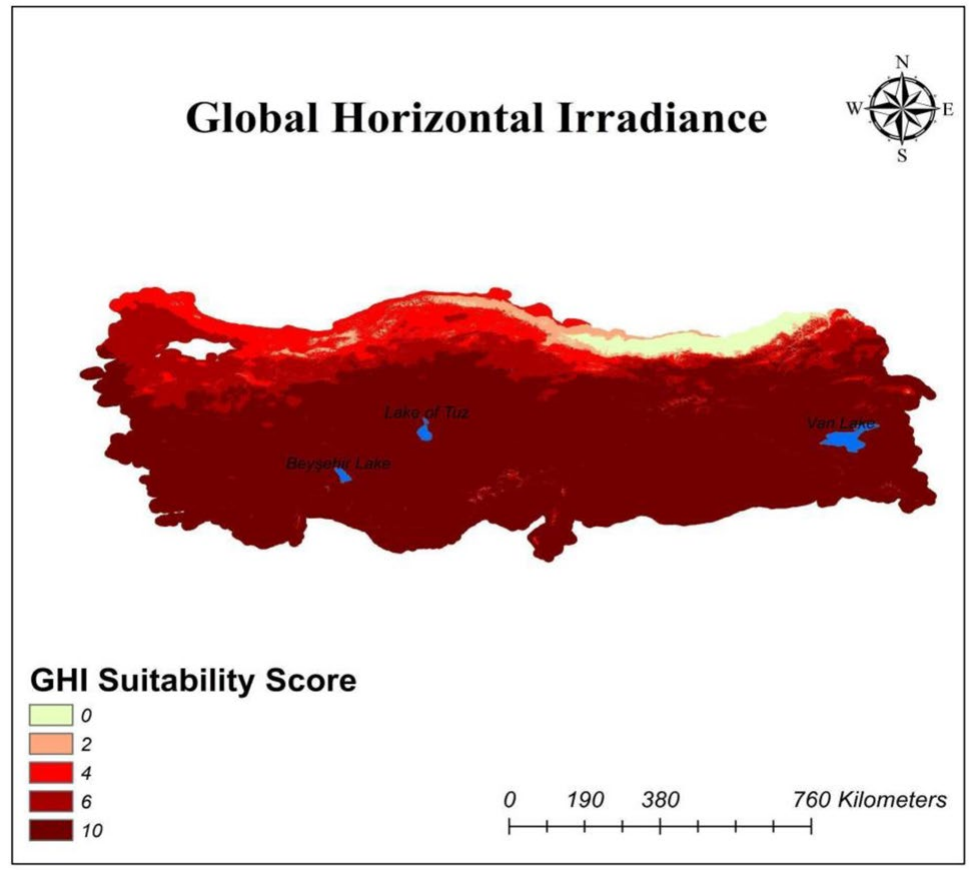
Global horizontal irradiance distribution map

The potential assessment for energy power plants is very critical. For the PV systems, GHI is the common input to assess the potential impact of sunlight (Nebey et al. 2020). In the study, the importance weight of GHI sub-criterion is provided in Table 8, and the assessment of different regions with respect to GHI is demonstrated in Fig. 2. Distribution score of GHI for study areas can be seen as similar to each other.

#### Distance from forest areas

**Fig. 3:**
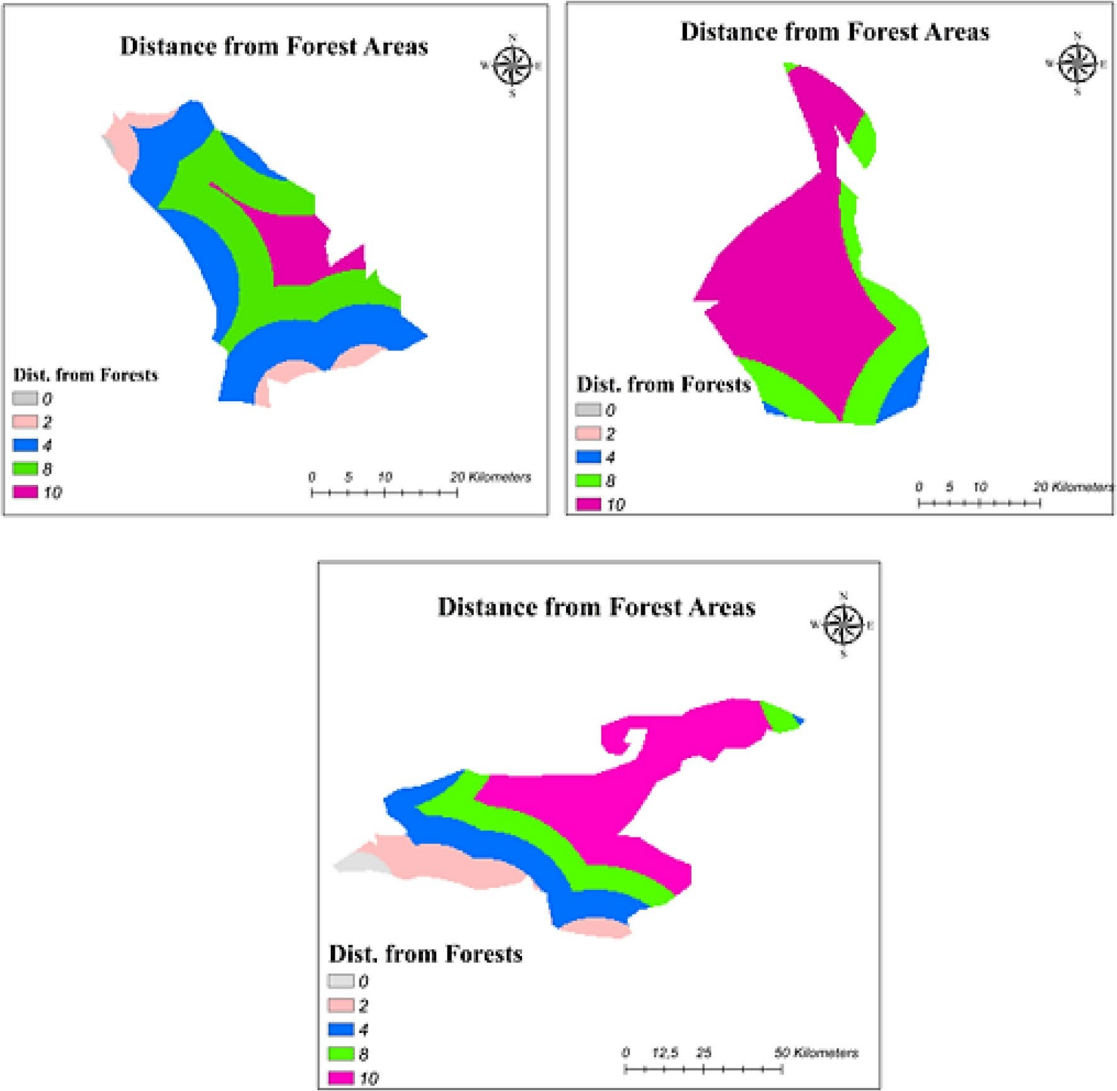
Distance from forest areas maps

One of the considerable sub-criteria for FPV systems was the distance from forest areas. Shadow effects of forests could affect solar performance negatively (Venkata 2007). Reclassification of distance from forest regions was determined considering maximum and minimum distance values. Suitability scores based on distance values are explained in Table 9, and suitability maps of study areas with respect to distance from forests are shown in Fig. 3.

#### Distance from main roads

**Fig. 4:**
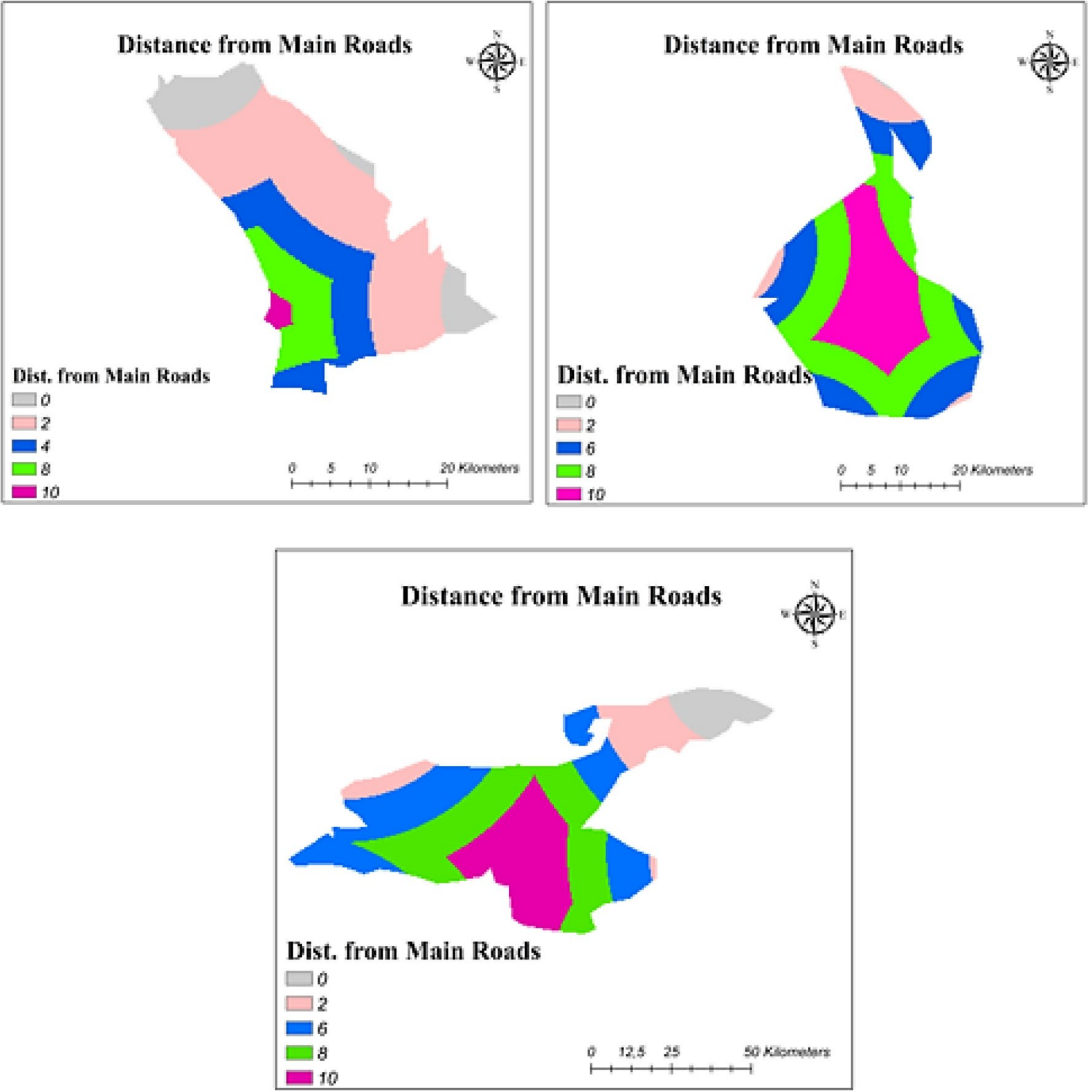
Distance from main road maps

Accessibility evaluation for energy power plants is very important from installation to maintenance-operation periods (Genç 2021). Therefore, the necessary data was taken from OpenStreet and proceeded with GIS. Suitability scores depending on distance from main roads are provided in Table 9, and maps of the lakes showing the distance from main roads are presented in Fig. 4.

#### Distance from main land

**Fig. 5:**
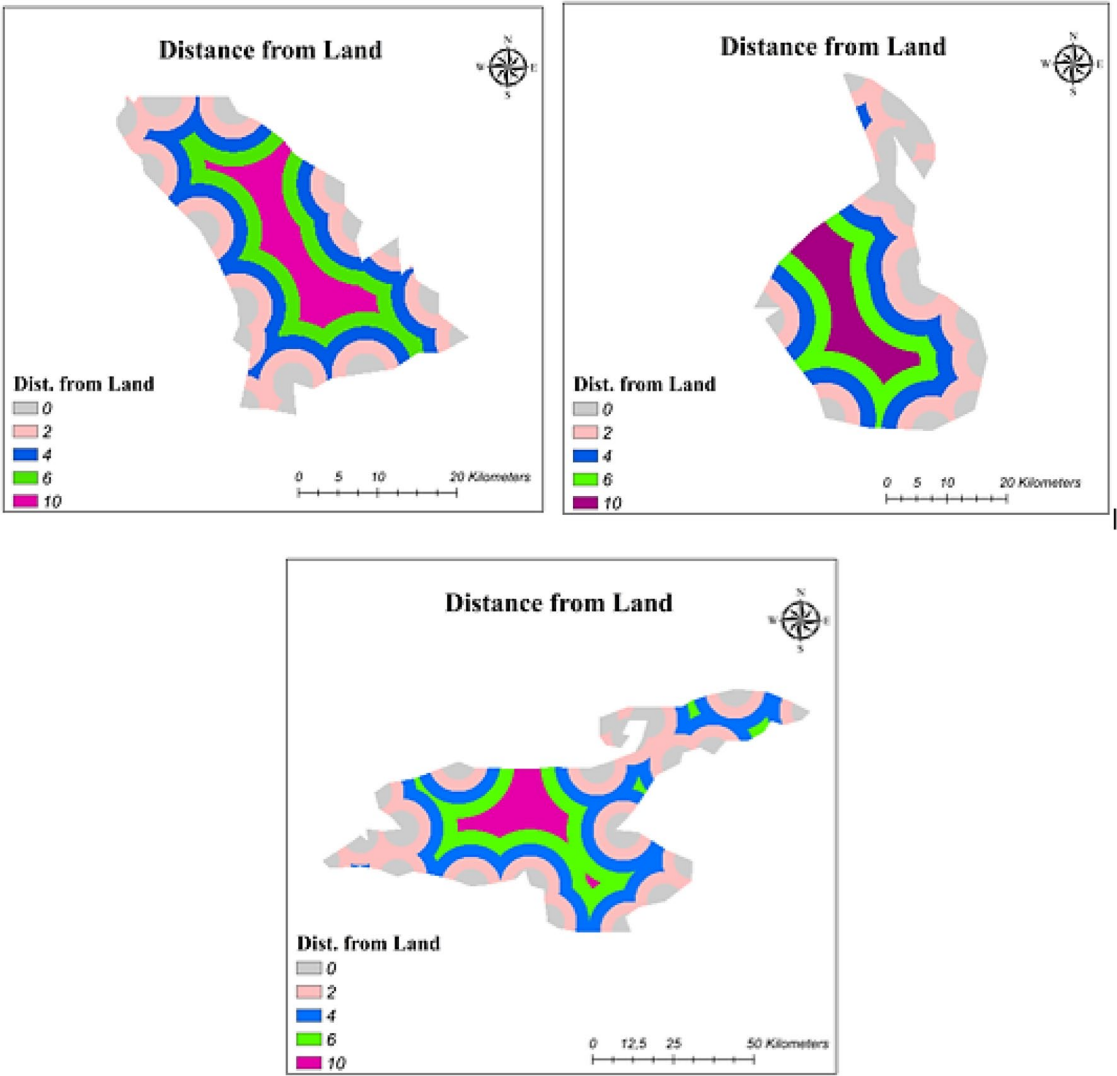
Reclassification of distance from land

Due to PV systems being high-cost investments and consisting of valuable products, these systems must be protected from threats. Although solar power plants were protected by using a fence on the land application, building a fence system for floating solar plants is difficult (Nebey et al. 2020). Therefore, FPV systems must be located at a certain distance to prevent the possible threats. The reclassification for distance from land is presented in Table 9, and maps showing the created layers based on distance from land are shown in Fig. 5.

#### Distance from electrical grid

**Fig. 6:**
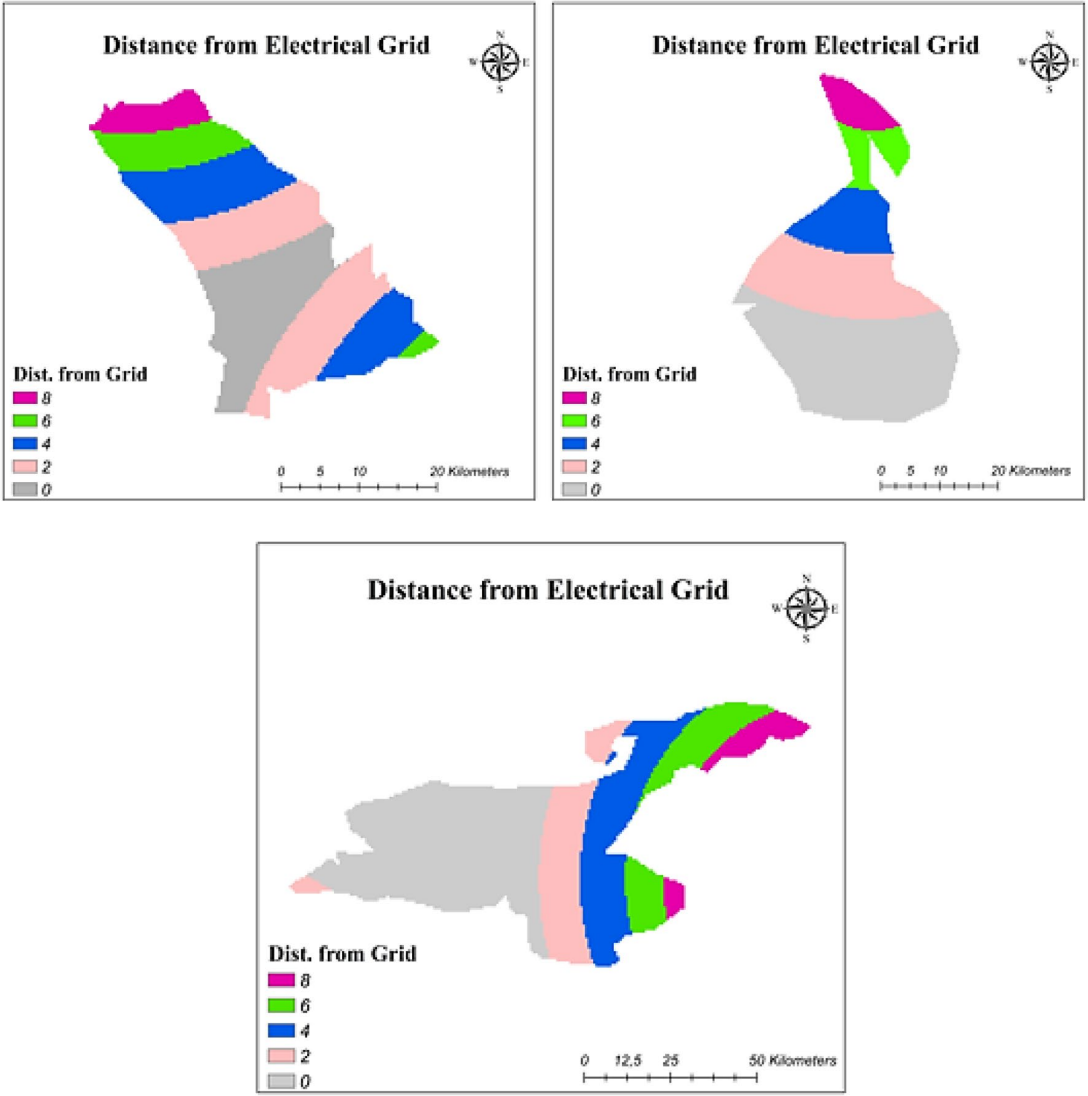
Reclassification of distance from electrical grid

Energy transmission processes are also significant for energy production. In the selection of appropriate areas for floating energy power plants, planning for energy transmission is a topic to work on. This study investigated the distance from the electrical grid to prevent high energy losses. Based on Turkey’s electricity grid data, suitability maps for three lakes are constructed as given in Fig. 6.

#### Distance from protected areas

**Fig. 7:**
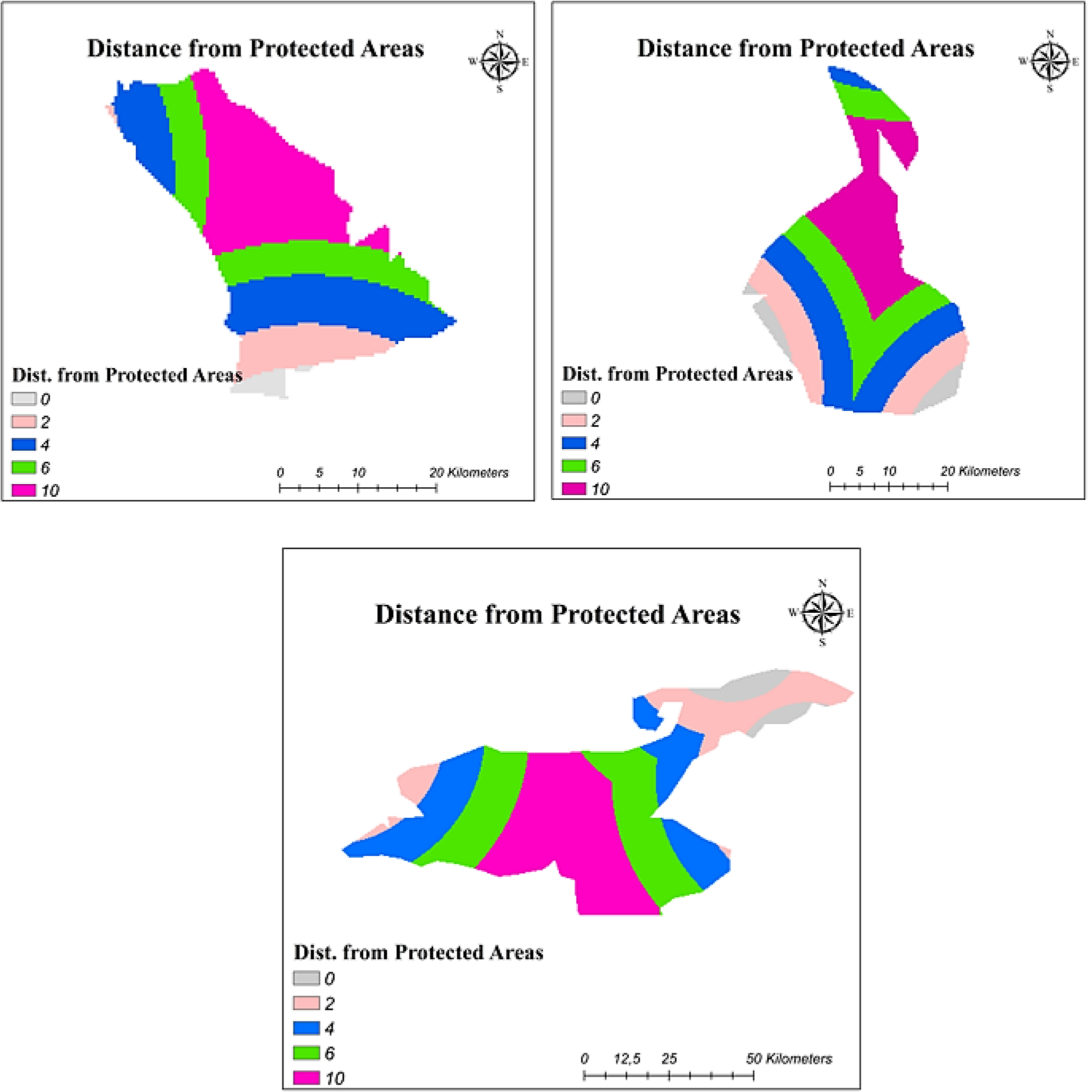
Distance from natural protected areas reclassified

Protected or designated areas, which were determined by national and international council, cannot use a different purpose (Genç et al. 2021). Turkey could be expressed as a rich country because of the presence of natural beauty. Therefore, the investigation of the effect of protected areas should be made. By using the protected areas in the vicinity of study areas, the reclassification process was completed and maps showing suitability index with respect to distance from protected areas are presented in Fig. 7.

#### Elevation

**Fig. 8:**
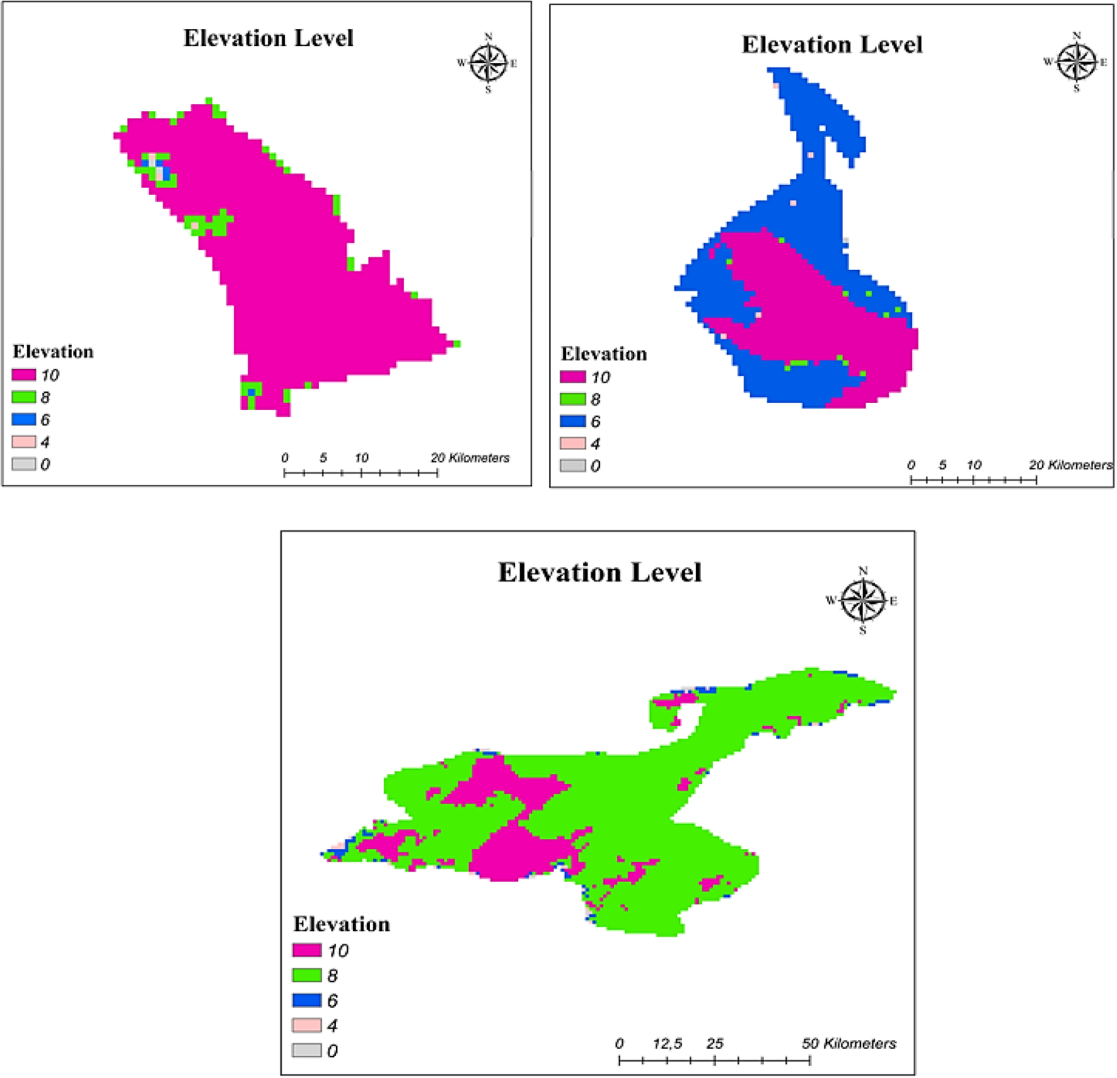
Elevation level reclassified

The assessment of the elevation levels for PV systems is necessary to provide high production performance. In the high levels of land, the temperature gets colder, causing the reduction in the energy produced with PV systems (Venkata 2007). In this study, the effect of elevation on PV systems was investigated as well. Reclassification based on elevation is indicated in Table 8, and maps showing suitability with respect to elevation are given in Fig. 8.

#### Human activities

This sub-criterion represents the possible negative impacts of humans on FPV. In Turkey, it is known that fishing on lakes and picnic activities around the lakes are common. Therefore, some security-related problems could be caused by humans who do other activities in the vicinity of the FPV.

#### Water pollution/marine effect

The FPV systems could cause water pollution during its lifetime (more than 20 years) due to its components such as metals, steel, and aluminum (Aman et al. 2015). It is known that water pollution negatively affects marine life which covers the lives of many species. Moreover, the installments of materials on the water surface and ground-mounted equipment can affect the spawning and breeding regions as well as private habitat area (Claus and Lopez 2022).

### Investigation of excluded restrictions

Activities with crowd participation, fishing, or picnic organizations at lakesides may negatively affect FPV systems for security reasons. As a result of the FAHP method, the weight of human activities restriction is found 0.033 which is quite low. The reason for this low weight is that adverse human activities towards PV systems are considered unlikely. Even though the authors intended to take this sub-criterion into account in the process of evaluating suitable regions for FPV systems, as they considered that buffer zones should be determined to prevent negative impact, the study had to exclude the human activities restriction from the resulting map due to lack of the assumption related to buffer distances.

**Fig. 9:**
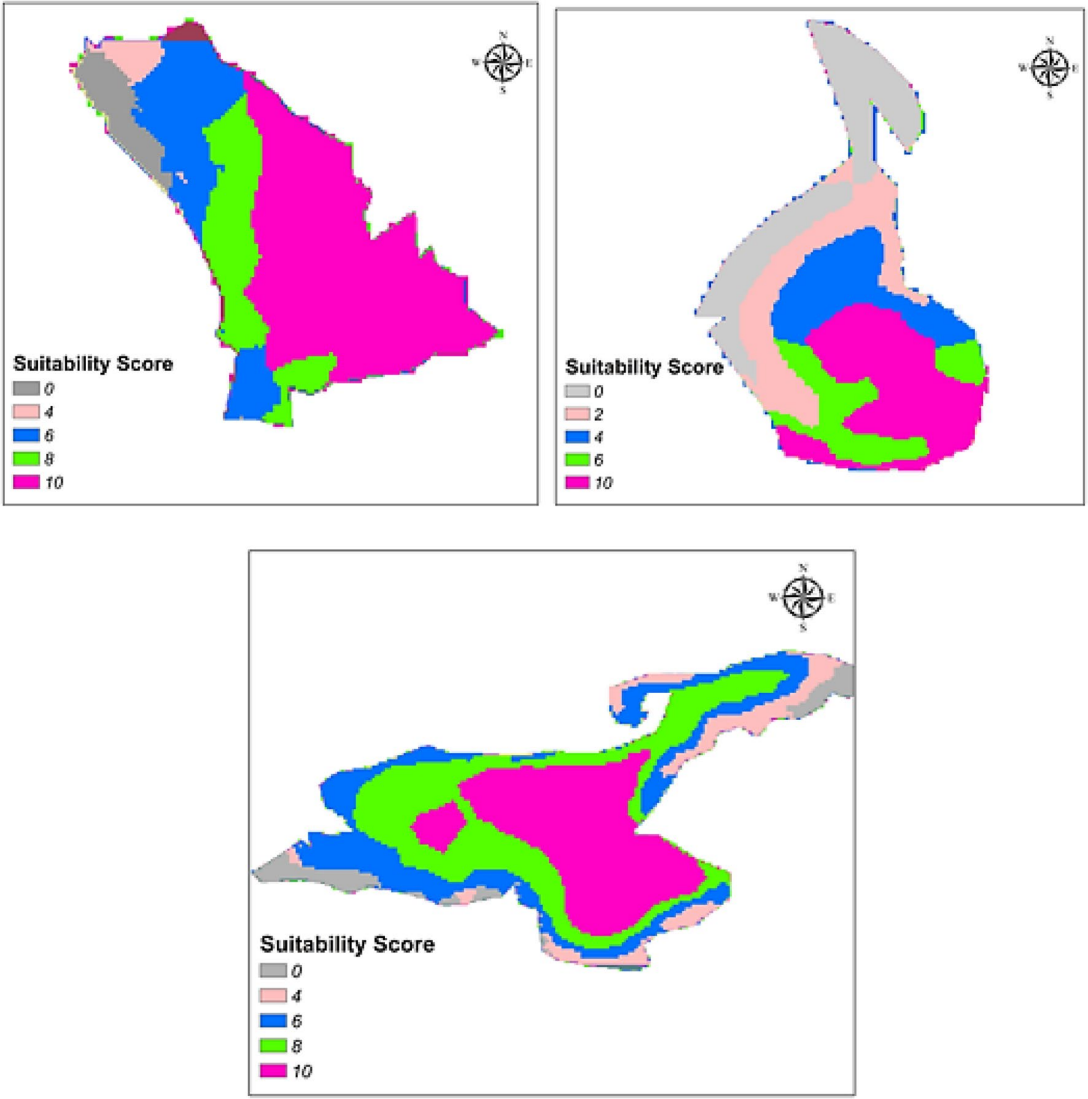
Overall suitability maps

In addition to human activities, the water pollution and marine effect can be considered as a critical point due to the mechanical structures of PV panels or montage equipment. According to FAHP results, the weight of this sub-criteria is 0.087. As with human activities, there was no assumption on buffer distance and there is no exact or approximate value on the effects of the FPV system on water pollution and marine life. The lack of information in this area caused this sub-criterion to be excluded from the study. These three lakes were evaluated using sub-criteria other than these two sub-criteria that had to be removed, and the maps given in Fig. 9 showing the overall suitability were obtained.

## Conclusions and future work

This study provided a methodology to determine the best location for the installment of FPV systems on lakes. The criteria set consisted of technical, environmental-social, and accessibility criteria with the identified ten sub-criteria. To measure the significance of each sub-criterion, the FAHP method was used. According to this MCDM structure, GHI constraint, which was a technical sub-criterion, was determined as the most important sub-criteria. Then, maps showing the evaluations of the study areas with respect to these sub-criteria have been created in ArcGIS software. By using the outputs of the FAHP method, which are sub-criteria weights, as inputs in GIS, the resulting maps showing the overall suitability layers for each study area were obtained.

According to maps showing overall suitability, the most appropriate lake for the installment of FPV systems has been determined as Beysehir Lake in terms of the proportion of the suitable area. As Turkey has a number of water bodies, the FPV systems are considerably important to produce the energy from renewables. Therefore, this study could be seen as a comprehensive guide to determine the most suitable regions for FPV system investments. For the regional assessments, the detected criteria set can change based on the requirements.

In addition to the findings above, the methodology itself is important in this study since after the prepared questionnaire was filled by asking the experts in this field, their answers were then examined with a comprehensive analytical model for better accuracy. Therefore, this allows researchers to take full advantage of the methodology and can bring this analytical model closer to many researchers for different problems.

In future studies, the excluded restrictions, human activities, and water pollution/marine effect will be investigated for the determined regions. To measure the potential impact of these restrictions, long-term observations and survey studies are planned.

## Data Availability

The datasets used during the present study are available from the corresponding author upon reasonable request.
